# Knowledge, attitude, and practice towards prevention of mother-to-child transmission of HIV among antenatal care attendees in Ethiopia, 2020

**DOI:** 10.1371/journal.pone.0277178

**Published:** 2023-02-24

**Authors:** Alex Yeshaneh, Haimanot Abebe, Fikremariam Endeshaw Tafese, Alemu Workineh

**Affiliations:** 1 Department of Midwifery, College of Medicine and Health Sciences, Wolkite University, Wolkite, Ethiopia; 2 Department of Public Health, College of Medicine and Health Sciences, Wolkite University, Wolkite, Ethiopia; 3 Saving Little Lives (SLL) Project, Hawassa University, Hawassa, Ethiopia; University of Tennessee Health Science Center College of Pharmacy Memphis, UNITED STATES

## Abstract

**Background:**

In 2020, globally approximately 37.6 million people living with HIV and 700,000 children are born infected from their parents. Every day there are nearly 1800 new Human Immune Virus (HIV) infections in children, more than 90% occurring in the developing world. Approximately 90% of these infections are associated with mother-to-child transmission (MTCT). In 2019, Ethiopia had over 100,000 pregnancies in HIV-positive women and over 12,000 HIV-positive. Therefore, this study aimed to assess the knowledge, attitude, and practice of pregnant mothers toward the prevention of mother-to-child transmission of HIV.ss

**Methods:**

An institutional-based cross-sectional study was conducted among 216 antenatal care (ANC) attendees in Gurage zone hospitals from June to July 2020. Data were collected using a structured and pre-tested questionnaire through face-to-face interviews. A Systematic random sampling technique with proportional allocation to size was used to select study subjects. Data entry and analysis were performed using Epi Data version 4.1 and SPSS version 25 respectively.

**Results:**

The level of good knowledge, attitude, and practice towards Prevention of mother to child transmission (PMTCT) of HIV among antenatal care attendees was found to be 72.2%, 79%, and 62% respectively. This study has also shown that at the time of transmission of the virus from the infected mother to her child, 38.9% of the respondents responded that it could be through breastfeeding, 38.9% during pregnancy, 16.5% during labor and 5.7% did not know respectively. All the respondents have been tested and 0.92% was positive. Nearly half, 99 (45.8%) of the respondents had tested for HIV/AIDS with their partner/husband during their ANC follow-up.

**Conclusion:**

In this study, the level of good knowledge, attitude, and practice towards PMTCT of HIV among antenatal care attendees were low. This finding also suggests that healthcare providers should consider the potential risk of mother-to-child transmission of HIV while providing clinical health assessments during antenatal care visits. Thus, improvement of counseling sessions and knowledge of PMTCT for pregnant women attending antenatal care is needed to increase their acceptance and use of PMTCT for HIV services.

## Introduction

Throughout history, mankind has observed and fought an invisible enemy of several pandemics; where a number of which were more disastrous to humans [1]. Acquired immunodeficiency syndrome (AIDS) is a chronic disease caused by the Human Immunodeficiency Virus (HIV) and continues as an epidemic [2]. The most common routes of HIV transmission include sexual contact, blood contact, and mother-to-child transmission (MTCT). MTCT is vertical transmission from HIV-positive pregnant women to their neonate during pregnancy, labor, delivery, or breastfeeding and is the most common mode of transmission in children [3, 4]. It’s succumbed to many people throughout the planet since it was first recognized in the early 1980s and no continent is spared of the pandemic [2].

Worldwide, 1.3 million pregnant women and 2.8 million children and adolescents are living with HIV. Despite significant efforts and achievements in PMTCT over the past decade, approximately 1.7 million children were living with HIV and 150,000 children were newly infected with HIV in 2019, mainly through the transmission of the virus from their mothers during pregnancy, delivery, or breastfeeding [5]. During this year, an estimated 95,000 children under the age of 15 died of AIDS-related causes globally and mainly in sub-Saharan countries [6]. In Ethiopia, the precise prevalence of HIV infection in children is unknown. However, 19,000 children ages 0–14 years are supposed to live with HIV/AIDS in urban areas [7].

Since 2011, the global community has committed itself to accelerating progress for the PMTCT initiative to eliminate new pediatric HIV infections and improve maternal, newborn, and child survival within the context of HIV [8]. Adherence to these practices is variable with better results obtained in developed countries than in developing countries [9]. Not surprisingly, the continuum of care, the magnitude of PMTCT, and associated services including HIV testing and counseling and ARV prophylaxis are still very low in developing countries [10, 11].

PMTCT of HIV has been considered one of the essential prevention interventions to control HIV/AIDS epidemics. Since the beginning of PMTCT programs, 1 million deaths and 2.2 million HIV infections have been averted among children [5]. One of the pillars of PMTCT and the most cost-effective way is to increase the knowledge, attitude, and practice of pregnant mothers toward this intervention strategy [12, 13].

Studies conducted in African countries showed that there was a low level of knowledge [14–22], attitude [14, 22–24], and practice [16, 25–30] of mothers regarding MTCT and PMTCT services. However, proper implementation of PMTCT services requires adequate knowledge and appropriate attitudes and practices of pregnant women toward PMTCT [14]. In the study area, data on knowledge, attitude, and practice toward PMTCT for HIV among pregnant mothers are not completely understood, and little is known about the benefits of PMTCT for HIV. Therefore, this study aimed to assess the knowledge, attitude, and practice toward PMTCT for HIV among pregnant mothers in the study area.

## Method and materials

### Study setting and design

An institutional-based cross-sectional study was conducted in Gurage Zone public hospitals from June- July / 2020. The Gurage zone is one of the 15 zones in south-central Ethiopia. It is 130 km from Addis Ababa the capital of Ethiopia, and 117 km from Hawassa the capital of the southern nation nationality and people region. The Zone had one specialized hospital, 1 general hospital, and 3 primary hospitals. These hospitals provide various services to their departments: under five Out Patient Departments (OPD) and adult OPD, Emergency OPD, Tuberculosis (TB) clinic, ART clinic, Maternal and child health services such as family planning, delivery, Abortion, Antenatal care (ANC) and PMTCT services.

### Population

#### Source population

All pregnant women attend ANC at Gurage Zone public hospitals.

#### Study population

Selected pregnant women attend ANC at Gurage zone public hospitals during the study period.

#### Inclusion and exclusion criteria

Pregnant mothers attending regular ANC follow-ups at Gurage zone public hospitals during the data collection period were included.

Mothers who were critically ill during the study period; clients with a documented history of mental illness and hearing impairment who were unable to provide the required information by themselves, pregnant mothers who started their ANC follow-up on the day of data collection, and those who were transferred from other health institutions were excluded.

#### Sample size determination

The required sample size was calculated using a single population proportion formula.


$$n=\frac{Z^{2}P(1-P)}{d^{2}}$$

Where;

**n** = sample size required

**Z** = standard normal distribution taken as 1.96 at 95% confidence level

d = margin of error taken as 5%

**P** = 84.3% proportion of respondents that have positive attitudes towards prevention of mother-to-child transmission of HIV in Mizan-Aman town, 2017) [22]. This proportion gives the highest Sample size and is considered for all objectives.

Thus; $n=\frac{1.96^{2}0.843(1-0.843)}{0.05^{2}}$ = 203

Adding 10% non-response rate the final sample was 223.

### Sampling techniques

Among the six hospitals found in the Gurage zone, three were selected using the lottery method (Wolkite University Specialized Hospital, Butajira General Hospital, and Sodo Buye Primary Hospital). A systematic random sampling technique with proportional allocation to the size was used to select the study unit. The sample was allocated proportionally to each hospital based on the number of ANC attendees at each hospital during the study period. The total number of ANC attendees on follow-up within the three hospitals during the study period was estimated to be 956. A total of 372 ANC attendees were followed up at the ANC clinic in the Wolkite University Specialized Hospital and 87 were selected for the study. There were 315 ANC attendees at Butajira General Hospital, of whom 73 were selected. Finally, 269 ANC attendees at Sodo Buye Primary Hospital, 63 were selected. The sampling interval was determined by dividing the expected number of ANC attendees per month into the sample size 956/223 which gives a sampling interval of four. Thus, every fourth coming to a follow-up service was interviewed until the total sample size was reached, using a systematic random sampling technique. The first case was selected using a lottery method at each hospital.

### Data collectors and Data collection procedure

To collect the data, five BSc Midwives were recruited as data collectors, and one public health officer supervised and coordinated the data collection process at each data collection sites. Following recruitment, training was provided about the data collection instruments in depth, and supervisors supervised the day to day data collection activity together with the principal investigator. Data were collected through face-to-face interviews using a structured and pre-tested questionnaire. The interview was conducted after the clients received the ANC service and each client was interviewed privately and assured of the confidentiality of the interview. Questionnaires were collected and checked for consistency every day by the investigators.

### Data collection instrument

Data was collected using a pre-tested interview-guided structured questionnaire. The questionnaire was prepared in English and translated into the local language and checked for consistency by translating it back to English by those who were well oriented with the stated languages (language Professionals or experts). The questionnaire contained: socio-demographic characteristics of the study respondents, knowledge, Awareness, and practice of the study respondents on PMTCT. The questionnaire was adapted from different studies developed for similar purposes by different authors to include all possible variables that addressed the objective of the study. For knowledge, attitude, and practice questions internal consistency of the items was checked using Cronbach’s alpha (α) and scored 0.81.

### Operational definitions

**Knowledge:** Knowledge was rated as: **Good** for those who answered>70% of knowledge questions correctly; **Fair**, for those who answer 50–70% of knowledge questions; and **Poor,** for those who answer<50% of the knowledge questions [31].

**Good attitude:** Those respondents who able to answer greater than or equal to 60% of the total attitude questions appropriately [32].

**Poor Attitude:** Those respondents answered less than 60% of the total attitude questions appropriately [32].

**Practice:** Practice is defined as the action of making the use of available PMTCT services such as testing of HIV, exclusive breastfeeding, use of ART prophylaxis, discussion about HIV with partner or husband, testing of partner or husband during ANC follow-up, getting of pre- and posttest counseling, participation in community conversation [31].

**Antenatal care: A c**are provided by skilled healthcare professionals to pregnant women to ensure the best health conditions for both mother and baby during pregnancy [33].

### Data quality assurance

To check the practicality and applicability of the questionnaire pretest was done on 5% of the sample size at Agena Primary Hospital. The English version of the questionnaire was translated into ‘guragegna’ and then back into English to maintain consistency. The final pretested and checked structured tool was used for data collection. Data were collected by personnel who had a health background and a BSc and supervised by an assigned supervisor and principal investigator. Prior to data collection, the research investigator provided 2 days of orientation to the data collectors regarding the objectives of the study, data collection techniques, and data collection tools. In addition, the principal investigator and supervisor gave feedback and corrections on a daily basis to the data collectors, and the completeness, accuracy, and clarity of the collected data were checked and reviewed carefully.

### Data processing and analysis

After data collection, the responses in the completed questionnaire were coded and entered into Epi Data version 4.1 and exported to Statistical Packages for Social Sciences (SPSS) version 25.0 for analysis. It was cleaned and edited (check for missing values and outliers) accordingly. Descriptive statistics were used to describe the frequency distribution, proportion, measures of central tendency, and dispersion. Finally, the result was presented using text, frequency tables, and graphs.

### Ethical consideration

Ethical clearance was obtained from the Institutional Review Board (IRB) of Wolkite University College of Medicine and Health Science. An official letter was sent to the Gurage Zone health office and the data collection was begun after obtaining a consent and cooperation letter. The study purpose, procedure, and duration, rights of the respondents and data safety issues, possible risks and benefits of the study were clearly explained to each participant using the local language. Participation information was collected anonymously after obtaining informed written consent from each respondent/parents/guardians by assuring confidentiality throughout the data collection period. In order to maintain confidentiality, the participants were assured that the obtained information is not made available to anyone who was not directly involved in the study and their names are not written on the questionnaire.

## Result

### Socio-demographic characteristics

A total of 216 women were included in this study, yielding a response rate of 97%. The mean age of the study participants was 28.6 (SD ± 5.4 years). Nearly all, 196(90.7%) of the respondents were married, and almost three-fourths, 165(76.4) were Gurage by ethnicity. Half, 108(50.0%) of the respondents had attended primary education. Regarding occupation, nearly four-fifths, 166(76.9%) of the participants were housewife. Half, 110(50.9%) of the participants had an average monthly income greater than 1500 ETB **(Table 1).**

**Table 1:** Socio-demographic characteristics of respondents in Gurage Zone public hospitals, Southwest Ethiopia, 2020.

Variables	Category	Frequency	Percent
Age in years	15–19	18	8.3
	20–24	61	28.2
	25–29	82	38.0
	30–34	27	12.5
	35–39	14	6.5
	> = 40	14	6.5
Marital status	Single	12	5.6
	Married	196	90.7
	Divorced	4	1.9
	Widowed	4	1.9
Ethnicity	Gurage	165	76.4
	Oromo	26	12.0
	Amara	21	9.7
	Tigre	2	0.9
	Others	2	0.9
Religion	Muslim	119	55.1
	Orthodox	63	29.2
	Protestant	34	15.7
Occupation	Housewife	166	76.9
	Government employ	20	9.3
	Merchant	16	7.4
	Privately employed	14	6.5
Educational status	Illiterate	38	17.6
	Primary (1–8)	108	50.0
	Secondary (8–12)	46	21.3
	College & above	24	11.1
Husband occupation	Farmer	39	19.9
	Government employ	61	31.1
	Merchant	77	39.3
	Privately employed	19	9.7
Monthly income	<500 ETB	15	6.9
	500–1000 ETB	41	19.0
	1000–1500 ETB	50	23.1
	>1500 ETB	110	50.9

### ETB: Ethiopian Birr

#### Reproductive history of respondents

Regarding gravidity, almost half, 110(50.9%) of respondents were gravida one and two. Concerning the parity status of the women, nearly two-fifths, 81 (37.5%) of the participants were para one and two. Almost one-third (35.6%) of the participants gave birth of their last child at the health center **(Table 2)**.

**Table 2:** Reproductive history of respondents in Gurage Zone public hospitals, Southwest Ethiopia, 2020.

Variables	Category	Frequency	Percent
Number of pregnancies experienced by the respondents	1–2	110	50.9
	3–4	73	33.8
	> = 5	33	15.3
Number of delivery experienced by the respondents	No delivery	59	27.3
	1–2	81	37.5
	3–4	44	20.4
	> = 5	32	14.8
Place of last delivery	Home	18	8.3
	Health center	77	35.6
	Hospital	54	25.0
	Other	8	3.7
	No delivery	59	27.3
Number of alive children	No child	59	27.3
	1–2	81	37.5
	3–4	44	20.4
	> = 5	32	14.8

#### Knowledge of respondents on PMTCT of HIV

**Fig 1:**
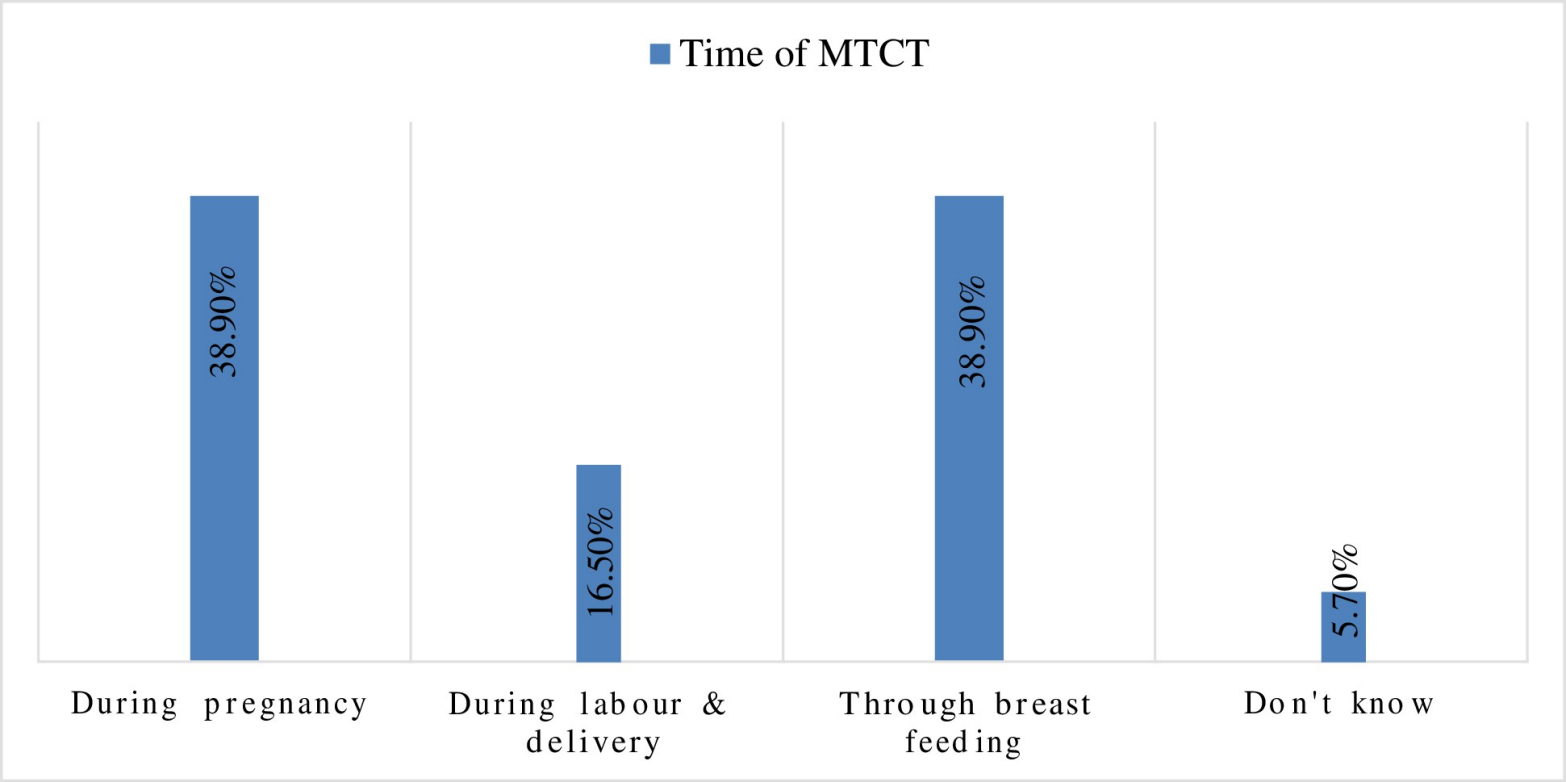
Knowledge of the respondents on the time of MTCT of HIV in Gurage Zone public hospitals, Southwest Ethiopian, 2020.

All of the respondents, 216 (100%) had heard of HIV/AIDS. The majority, 195(90.3%) of respondents had heard about MTCT of HIV. Nearly two-fifth, 76(38.9%) of the respondents responded that HIV could be transmitted from the infected mother to her child through breastfeeding, 76(38.9%) of the respondents responded during pregnancy, 32(16.5%) of the respondents responded during labor and delivery and 11 (5.7%) of the respondents were responded as they were not familiar with the transmission period of MTCT of HIV **(Fig 1)**.

The majority, 190(87.96%) of the respondents had heard about the prevention method of MTCT of HIV. Nearly three-fifths, 112(58.9%) of the respondents knew safer infant feeding, 120(63.2%) of the respondents knew ART drugs, 103(54.2%) of the respondents knew safe delivery practice, 63(33.2%) of the respondents knew educating the community about risky behaviors and promoting condom use, 60(36.3%) of the respondents knew one-to-one HIV counseling, testing and treatment of STI and, 59(31.1%) of the respondents knew prevention of unintended pregnancy by improving FP service given for HIV-positive pregnant mothers could reduce the risk of HIV transmission **(Table 3)**.

**Table 3:** Knowledge of MTCT of HIV/AIDS and prevention method of MTCT of HIV in Gurage Zone public hospitals, Southwest Ethiopia, 2020.

Variables	Category	Frequency	Percent
Heard about MTCT of HIV	Yes	195	90.3
	No	21	9.7
Heard about PMTCT of HIV	Yes	190	87.96
	No	26	12.04
Safe delivery practice to reduce the risk MTCT of HIV	Yes	103	54.2
	No	87	45.8
Safe infant feeding practice to reduce the risk MTCT of HIV	Yes	112	58.9
	No	78	41.1
HIV counseling, testing, and treatment of STI to reduce the risk MTCT of HIV	Yes	60	36.3
	No	130	68.4
ART service to reduce the risk MTCT of HIV	Yes	120	63.2
	No	70	36.8
Prevention of unintended pregnancy by improving FP service to reduce the risk MTCT of HIV	Yes	59	31.1
	No	131	68.9
Education of the community about risky behaviors and promote condom use to reduce the risk of MTCT of HIV	Yes	63	33.2
	No	127	66.8
Follow up on ART clinic for baby born from HIV-positive mother	Yes	161	74.5
	No	55	25.5

Nearly three-fourths, 161(74.5) of the respondents knew that babies born from HIV-positive mothers needed to follow up on ART clinics. Regarding the duration of follow-up, 133 (82.6%) of the respondents responded that a child needs follow-up until proven HIV negative, 21(13.0%) of the respondents responded that a child needed to follow up for six months, and 7 (4.4%) of the participants responded that a child need follow up for one year.

**Fig 2:**
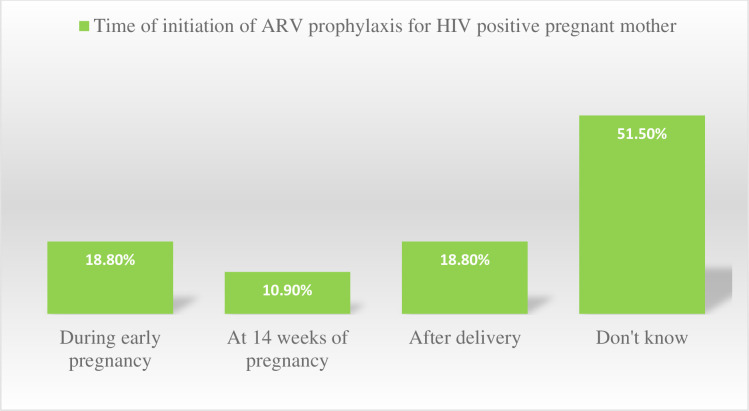
Knowledge of the respondents on time of initiation of ARV prophylaxis in Gurage Zone public hospitals, Southwest Ethiopian, 2020.

The majority of the respondents, 130(60.2%) knew that it was possible to reduce the risk of HIV transmission if an HIV-positive mother decided to breastfeed her baby. Eighteen, (13.8%) the respondents knew about exclusive breastfeeding, 47(36.2%) the respondents knew early weaning with formula, and 64(49.2%) the respondents knew taking ARV prophylaxis could reduce the risk of HIV transmission through breastfeeding if an HIV-positive mother decided to breastfeed her baby. This study revealed that, almost half, 33(51.5%) of the respondents do not knew the time of ART prophylaxis initiation **(Fig 2)**.

**Fig 3:**
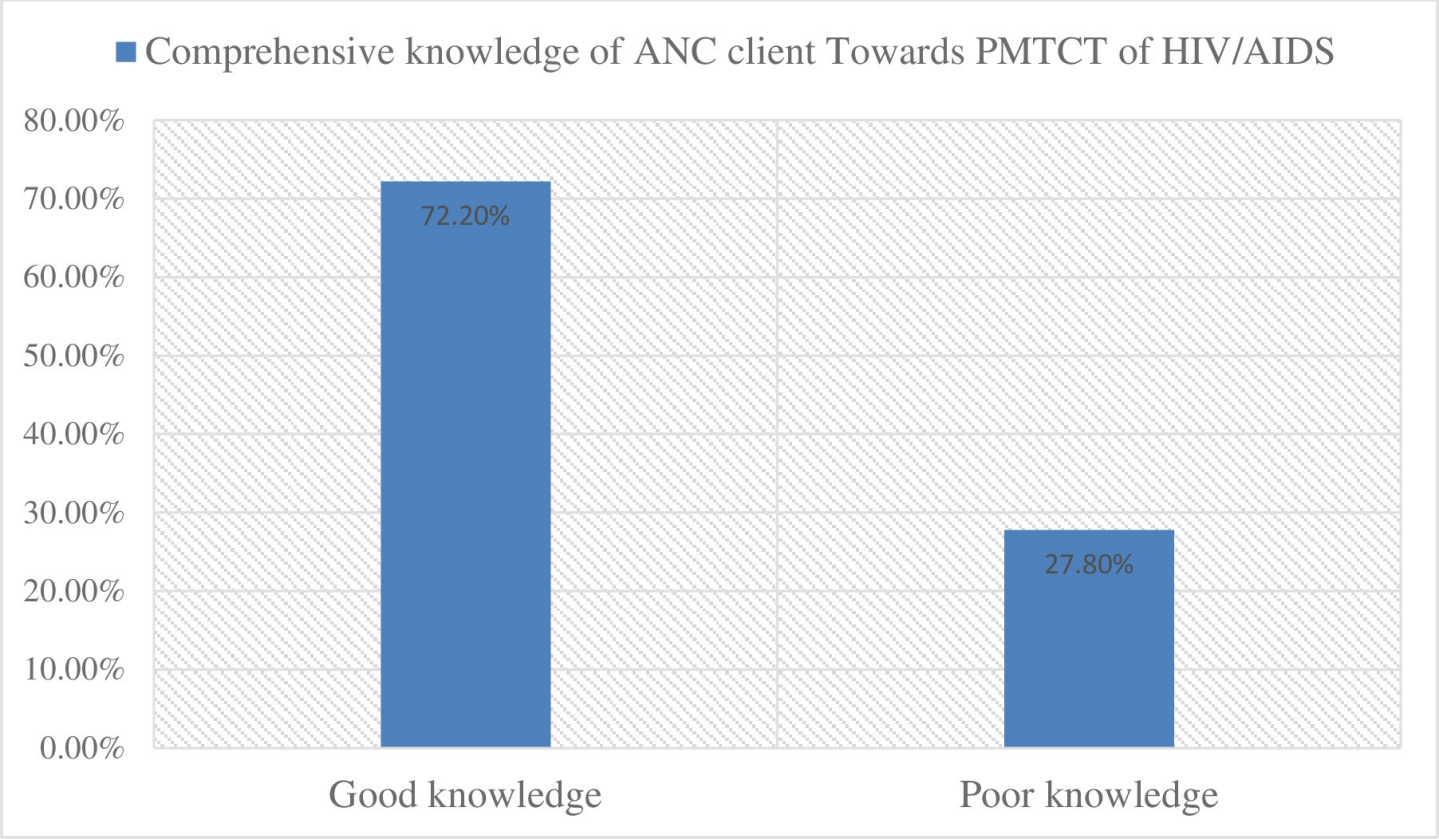
Comprehensive knowledge of respondents towards PMTCT of HIV in Gurage Zone public hospitals, Southwest Ethiopia, 2020.

The comprehensive knowledge regarding PMTCT of HIV of respondents had 72.2% good knowledge **(Fig 3).**

### Attitudes of ANC clients regarding PMTCT of HIV

The majority, 194 (89.8%) of the respondents believed that HIV can be transmitted from mother to child. Nearly three-fourth, 159 (73.6%) of respondents had agreed that MTCT of is preventable. Almost all, 206(95.4%) of the participants accepted the idea of knowing their HIV status. The majority, 195(90.3%) of the respondents were agreed to tell for their husband if they became HIV-positive. Nearly four-fifths, 165(76.4%) of the respondents did not encourage condom use with spouses. Almost three-fourths, 161(74.5%) of the respondents were agreed with the idea that HIV-positive women can have a baby **(Table 4).**

**Table 4:** Attitude of ANC clients regarding PMTCT of HIV respondents in Gurage Zone public hospitals, Southwest Ethiopia, 2020.

Variables	Category	Frequency	Percent
HIV can be transmitted from mother to child	Yes	194	89.8
	No	22	10.2
MTCT of HIV is preventable	Yes	159	73.6
	No	57	26.4
pregnant mother should tell her test result to her husband	Agree	195	90.3
	Disagree	21	9.7
Condom use with spouse	Encourage	51	23.6
	Not encourage	165	76.4
HIV-positive women can have baby	Yes	161	74.5
	No	55	25.5
Breast milk is nutritionally complete	Agree	190	88.0
	Disagree	14	6.5
	Neutral	12	5.5

**Fig 4:**
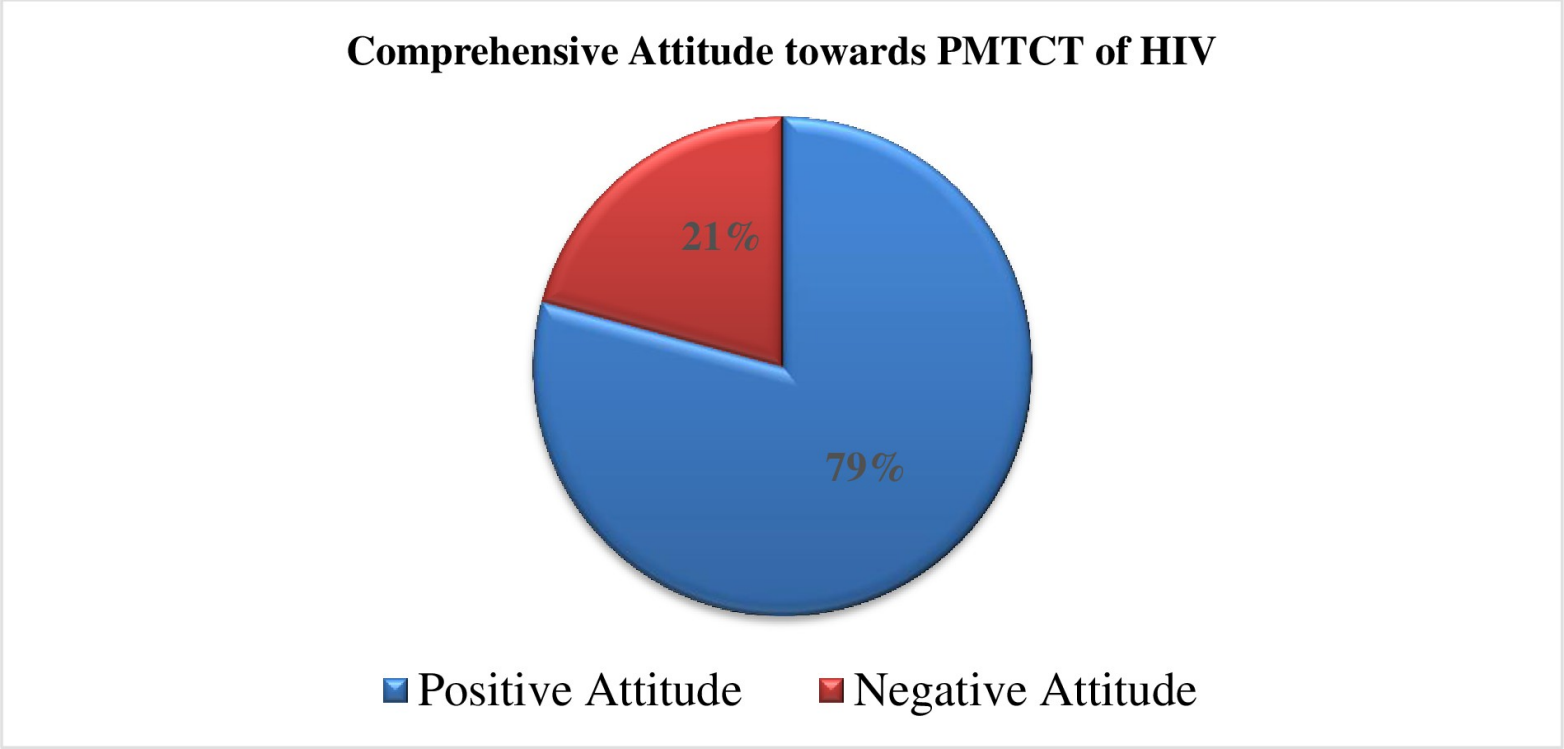
Comprehensive attitude of ANC client towards PMTCT of HIV in Gurage Zone public hospitals, Southwest Ethiopian, 2020.

The overall attitude of ANC clients regarding PMTCT of HIV, 171 (79.2%) of the respondents had good attitudes toward PMTCT of HIV **(Fig 4).**

### Practices of ANC clients regarding PMTCT of HIV

All of the respondents, 216(100%) have been tested for HIV and 99% had negative serostatus. Almost two-fifths, 88 (40.7%) of the respondents had received pre- and post-testing counseling. All of the respondents, 214(99.1%) were willing to have an HIV test in the current pregnancy. Almost all of the respondents, 212(99.0%) had the willingness to accept their test results. Almost one-third, 76(35.9%) of respondents were not willing to disclose their test results. Nearly half, 99 (45.8%) of the respondents had tested for HIV/AIDS with their partner/husband during their ANC follow-up. The majority, 199(92.1%) of the respondents had no participation in community conversations on HIV/AIDS. Three-fifths, 134(62%) of the respondents had good practice toward PMTCT of HIV **(Table 5).**

**Table 5:** Practices of ANC client on PMTCT of HIV/AIDS HIV in Gurage Zone public hospitals, Southwest Ethiopia, 2020.

Variables	Category	Frequency	Percent
Ever been tested for HIV	Yes	216	100
Sero-status	Positive	2	0.92
	Negative	214	99.08
Pre and post-test counseling	Yes	88	40.7
	No	128	59.3
ANC follow-up in a current and previous pregnancy (Hint: ANC follow-up preceding day of the survey)	Yes	141	89.8
	No	16	10.2
Willing to have HIV test in current pregnancy	Yes	214	99.1
	No	2	0.9
Willing to accept test result	Yes	212	99.0
	No	2	1.0
Willing to disclose test result	Yes	76	35.9
	No	136	64.1
Husband or partner tested for HIV during her ANC follow up	Yes	99	45.8
	No	117	54.2
Participated in a community conversation on HIV/AIDS	Yes	17	7.9
	No	199	92.1

## Discussion

The study revealed a variety of concerns relating to the level of knowledge, attitude, and practices associated with the use of PMTCT programs at public health facilities in Gurage Zone. As for the part of the ANC service, the analysis showed key options for PMTCT prevention strategies. Similarly, in order to introduce comprehensive HIV responses such as ARV prophylaxis for children born to HIV-exposed mothers, to initiate ART drugs for mothers based on choice B+ rules, and to prevent sustained pregnancies, it is important to improve awareness of HIV, MTCT, and PMTCT among populations at higher risk of HIV infection.

In this study, 72.2% of ANC clients had good knowledge about PMTCT of HIV. The findings in this study showed that there is indeed an established network in the study area for ANC participants, and this could serve as a starting point to produce a shared ANC client in PMTCT in the study setting. This proportion was much lower than a study from China, and Gondar [34, 35]. The methodology applied and research environments, socio-demographic attributes of the participants, and the quality and availability of health service infrastructures may be the reason for the difference.

This result suggests that there is a difference that, in partnership with the local health care provider, the district health office and regional health office should collaborate to increase the knowledge in the prevention of mother-to-child transmission of HIV.

This proportion, however, was significantly higher than that of Tanzania and South Africa [17, 26]. The discrepancy may probably be explained by the difference in better attention has been given to PMTCT these days, the time gap and changes in health care systems contribute to good knowledge of PMTCT.

Regarding the attitude of respondents, 79% of them had a positive attitude towards PMTCT. This finding is higher than a study conducted in Kenya and Mizan Aman Ethiopia [22, 23]. The possible reason could be the change in the health care system because the Ethiopian government has put great attention on maternal and newborn health, a difference in the quality of service offered to mothers’ expectations, or the nature of health care facilities. Simultaneously, the explanation for this disparity may be due to the comprehensive work which is done by health extension personnel and various health care organizations in raising awareness of the research areas about PMTCT and the study time gaps, methodological concerns, and society’s cultural context may contribute for the difference. However, this finding is also lower than a study done in South Africa and South Ethiopia [14, 19]. The reason could be expressed by the difference in applying policies for the prevention of MTCT of HIV between countries, study period, study setting, time gap, methodological differences, and socio-demographic discrepancy of the respondents. Similarly, the justification might be attributed to the strategies implemented on maternal and child health, PMTCT, and other women empowering policies by the government as well as other non-governmental organizations in the study setting.

Concerning the practice of women towards PMTCT of HIV, 62% of them had good practice of PMTCT. This report is slightly higher than studies conducted in South Africa, Togo, and Arbaminch Ethiopia [26–27, 30]. This may be explained by the difference in the study setting, study design, study period, methodological differences, and socio-cultural background disparity between the study subjects and in these days the government of Ethiopia and any non-governmental organization has emphasized PMTCT of HIV to overcome the distribution of mother to child transmission of HIV.

On the other side, this finding related to the practice of ANC clients towards PMTCT of HIV is less than a study done in Nigeria and Hosanna Ethiopia [29, 31]. The reason might be explained by intervention given for maternal and child care practices including the provision of PMTCT of HIV in maternal and child health service clinics by the concerned bodies could be attributed to this difference. In the same way, this discrepancy might also be related to variation in women’s educational status and sample size variation, the background of the study participants and method used and study settings, and the availability and accessibility of the infrastructures. In nutshell, this finding implies that there is a lack of balance that the zone health office regional health bureau, and other organization that works on HIV/PMTCT could work in collaboration with the local health care provider to enhance the good practice of women towards PMTCT of HIV in ANC clinics. Overall, the knowledge, attitude, and practice toward PMTCT of HIV were lower as compared to the majority of previous studies [14, 19, 29, 34]. This might be due to the primary educational level (>50%), housewife in occupation (76.9%), low income (49.1%), and knowledge gap on HIV counseling, testing, and treatment of STI to reduce the risk MTCT of HIV, prevention of unintended pregnancy by improving FP service to reduce the risk MTCT of HIV and not encouraging condom use with spouse due to culture in this study were high as compared to other previous findings.

### Limitations of the study

This study shares the limitation of study design and our study was not a mixed type of study, it couldn’t address some important variables and cause-effect relationships. In depth interview could more dig the problem and this study couldn’t addressed it.

## Conclusion

In this study, the level of knowledge, attitude, and practice towards PMTCT of HIV among antenatal care attendee was found to be low. This finding also suggests that health care providers should take into account the potential risk of mother-to-child transmission of HIV while giving clinical health assessments during antenatal care visits. Besides, for healthcare planners this is vital. These findings can be used to build relevant programs, channeling scarce resources to teaching what is needed as opposed to imparting messages that are already known. Thus, improvement of counseling sessions for pregnant women attending ANC at Gurage Zone hospital is needed to increase their acceptance and use of PMTCT of HIV services.

## Supporting information

S1 File This S1 File minimal data set of KAP on PMTCT. (SAV)

## List of abbreviation

ANC: Antenatal Care
ART: Anti Retro Viral Treatment
ETB: Ethiopian Birr
IRB: Institutional Review Board
HAART: Highly Active Antiretroviral Therapy
HIV: Human Immune Virus
MTCT: Mother to Child Transmission
OPD: Out-Patient Department
PMTCT: Prevention of Mother to Child Transmission
SPSS: Statistical Packages for Social Sciences
